# Improving the Precision and Speed of Euler Angles Computation from Low-Cost Rotation Sensor Data

**DOI:** 10.3390/s150307016

**Published:** 2015-03-23

**Authors:** Aleš Janota, Vojtech Šimák, Dušan Nemec, Jozef Hrbček

**Affiliations:** Department of Control & Information Systems, Faculty of Electrical Engineering, University of Žilina, Univerzitná 8215/1, Žilina 010 26, Slovakia; E-Mail: vojtech.simak@fel.uniza.sk (V.Š.); nemec.dusan666@gmail.com (D.N.); jozef.hrbcek@fel.uniza.sk (J.H.)

**Keywords:** gyroscope, Euler angle, inertial navigation, MEMS, rotational matrix

## Abstract

This article compares three different algorithms used to compute Euler angles from data obtained by the angular rate sensor (e.g., MEMS gyroscope)—the algorithms based on a rotational matrix, on transforming angular velocity to time derivations of the Euler angles and on unit quaternion expressing rotation. Algorithms are compared by their computational efficiency and accuracy of Euler angles estimation. If attitude of the object is computed only from data obtained by the gyroscope, the quaternion-based algorithm seems to be most suitable (having similar accuracy as the matrix-based algorithm, but taking approx. 30% less clock cycles on the 8-bit microcomputer). Integration of the Euler angles’ time derivations has a singularity, therefore is not accurate at full range of object’s attitude. Since the error in every real gyroscope system tends to increase with time due to its offset and thermal drift, we also propose some measures based on compensation by additional sensors (a magnetic compass and accelerometer). Vector data of mentioned secondary sensors has to be transformed into the inertial frame of reference. While transformation of the vector by the matrix is slightly faster than doing the same by quaternion, the compensated sensor system utilizing a matrix-based algorithm can be approximately 10% faster than the system utilizing quaternions (depending on implementation and hardware).

## 1. Introduction

Micro-Electro-Mechanical systems (MEMS) represent the integration of mechanical elements, sensors, actuators, and electronics on a common silicon substrate through the utilization of microfabrication technology [1]. The number of MEMS used in various applications is permanently growing due to the small dimensions, light weight, lower power consumption, higher reliability, and relatively low cost which makes them commercially available. Typical MEMS-based low-cost products are accelerometers, gyroscopes, pressure sensors, microphones, digital mirror displays, micro pumps, *etc.* For the purpose of low-cost navigation solutions MEMS-based inertial sensors (accelerometers and gyroscopes) have been developed since orientation of an object in the three-dimensional space is key information needed for navigation, guidance and control tasks. MEMS inertial sensors may be found in variety of applications from traditional ones (navigation and positioning of various transport means and/or robots) to sensing of human body walking and movement [2,3,4,5], daily life surveillance [6] or new commercial applications available through smart phones [7]. Most studies on MEMS gyroscopes are focused on their performance, and common methods to improve the performance [8]. Unlike non-micro devices MEMS sensors experience more errors that build up over time, corrupting the precision of the measurements and eventually rendering the navigation solution useless [9,10]. Thus the first and easiest-to-measure performance criterion of a gyro is its static readout as a function of time. Accuracy is usually limited by electrical noise, systematic errors and/or mechanical thermal noise [11,12]. The static compensation of sensor inaccuracies can be enabled by proper calibration methods designed for MEMS gyroscopes and accelerometers [13]. The principle of recently developed micro-machine gyroscopes, their structures and classification can be found in [14].

Generally, gyroscopes measure rotational rate, which can be integrated to yield changes in orientation. An effective method most used to parametrize the orientation space is based on usage of so called Euler angles. Euler angles are used as a framework for formulating and solving the equations for conservation of angular momentum. This article has been written with motivation to analyze and show how precision and speed of computations of Euler angles could be improved when processing data from the MEMS gyroscope. It is organized as follows: Section 1 (Introduction) describes theoretically several methods of notation to express rotation of a body (particularly the rotation matrix, Euler angles, rotation around arbitrary axis, and quaternion). Section 2 (Experimental Section) is focused on comparison of errors occurring when algorithms utilizing described notations process data from the gyroscope. If applicable, more versions of the same algorithm are considered (focused either on accuracy or fastness of computation). At the end of the section there is discussion on how errors presented in real gyroscopes could be compensated. Section 3 (Results and Discussion) summarizes analyzed properties and gives final comparison and overview of obtained results. Finally, Section 4 gives the conclusions. The article is an extended version of the conference paper [15], elaborated and supported by the VEGA1/0453/12 grant and used with kind permission of Springer Science + Business Media. Article extensions resulted from the work under another project as stated in the Acknowledgments section.

The purpose of the inertial navigation in the 3D space is to determine six independent variables: translation of an object in three axes and its rotation in three axes, relative to the inertial frame of the reference body. In this article we describe possible ways how to express rotation (attitude) of the object and calculate it from angular velocity measured by the gyroscope.

**Figure 1:**
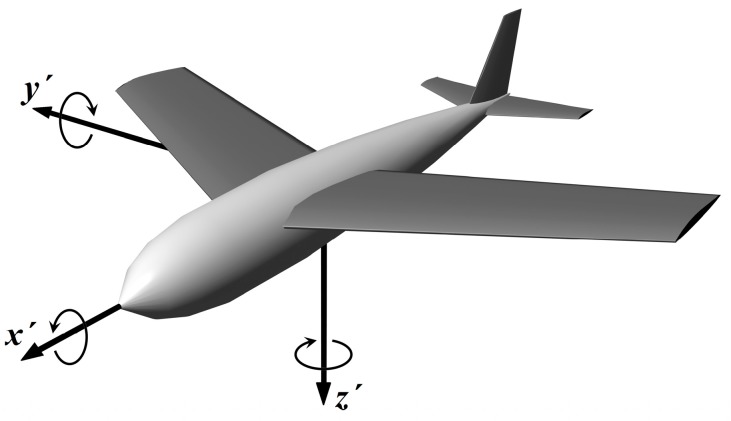
Orientation of the coordinate system axes.

We consider the Cartesian (orthonormal) right-hand coordinate system oriented by convention NED, *i.e.*, North-East-Down. Moving object axes’ orientations are *x* → forward, *y* → right and *z* → down (Figure 1). Two reference frames are used:

- Frame of reference joined with Earth (considered to be approximately inertial), marked *S.* All variables measured with respect to Earth will be marked without a dash.
- Frame of reference joined with rotating object, marked S’. All variables measured onboard the moving object will be marked with a dash.

First we will analyze four used methods of notation that allow us to express rotation of a body. Differences among those individual approaches can be seen in data redundancy and consumption of computer time during processing of raw data from the gyroscope and during conversion from one notation to another (which has direct impact on algorithm efficiency).

### 1.1. Euler Angles

**Figure 2:**
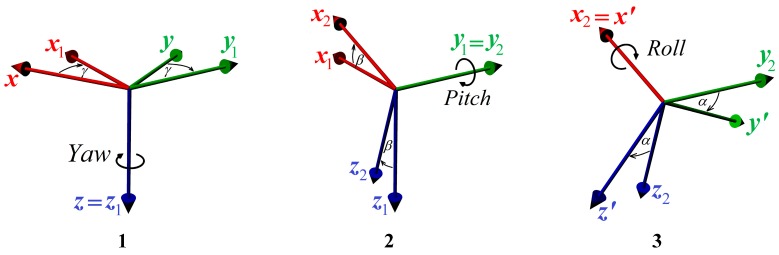
Euler angles for 3-2-1 convention.

Euler angles are expressing rotation of the object as a sequence of three rotations around objects’ local coordinate axes. This way of rotation expression is most interpretative and has zero data redundancy because only three real numbers are needed. Different sequence of axis rotation produces different resultant rotation; therefore Euler angles are defined according to chosen sequence (convention). In aviation the most used convention is *z-y-x* convention (sometimes called Yaw-Pitch-Roll convention or 3-2-1, see Figure 2):

1. Rotate the object around its *z*-axis by angle Yaw (marked *γ*);
2. Rotate the object around its new *y*_1_-axis by angle Pitch (marked *β*);
3. Rotate the object around its new *x*_2_-axis by angle Roll (marked *α*).

Rotation order of *z-y-x* convention can be expressed by the following operator:

(1) $${ℜ}_{\alpha ,\beta ,\gamma }={ℜ}_{\alpha }^{x_{2}}\left({ℜ}_{\beta }^{y_{1}}\left({ℜ}_{\gamma }^{z}\right)\right)$$

Inverse rotation is given by the reversed rotation order by inverted angles:

(2) $${ℜ}_{\alpha ,\beta ,\gamma }^{-1}={ℜ}_{-\gamma }^{z_{1}}\left({ℜ}_{-\beta }^{y_{2}}\left({ℜ}_{-\alpha }^{x^{\prime }}\right)\right)$$

Main disadvantage of representing object’s rotation by Euler angles is a lack of the simple algorithm for vector transformation. This can be realized by transferring Euler angles to the rotation matrix by Equation (10) and following application of Equation (4). Trivial chaining (adding) of two rotations represented by Euler angles is not possible.

### 1.2. The Rotational Matrix

The rotation matrix defines change of coordinates of the object in the coordinate system *S* during rotational movement. It is a typical representation of object’s attitude (very often used, e.g., in computer graphics). It is clear that this form has the greatest data redundancy due to needs of saving nine real numbers:

(3) $$R=\left[\begin{array}{ccc} R_{11} & R_{12} & R_{13}\\ R_{21} & R_{22} & R_{23}\\ R_{31} & R_{32} & R_{33} \end{array}\right]$$

Transformation of coordinates from the system *S* to the system *S'* can be done by multiplication of the position column vector ***r*** by the rotation matrix:

(4) $$r^{\prime }=R\cdot r$$

Result of rotation ***R***_1_ followed by ***R***_2_ is given by matrix multiplication:

(5) $$R=R_{2}\cdot R_{1}$$

Inverse rotation is given by the transposed matrix:

(6) $$R^{-1}=R^{T}$$

While the original vector ***r*** has the same length as the resultant vector ***r****'*, the rotational matrix has to be orthogonal with its determinant equal to 1. The matrix is orthogonal when all its row or column vectors are perpendicular to each other. The following algorithm can be used to normalize the matrix to be pure rotational [16]:

1. Calculate deviations *e_ik_* from orthogonality of the matrix columns:
  (7) $$X=\left[\begin{array}{c} R_{11}\\ R_{21}\\ R_{31} \end{array}\right]\;Y=\left[\begin{array}{c} R_{12}\\ R_{22}\\ R_{32} \end{array}\right]\;Z=\left[\begin{array}{c} R_{13}\\ R_{23}\\ R_{33} \end{array}\right]\;\begin{array}{c} e_{xy}=X\cdot Y\\ e_{yz}=Y\cdot Z\\ e_{zx}=Z\cdot X \end{array}$$
2. Distribute errors among all columns:
  (8) $$\begin{array}{l} X_{\text{ort}}=X-\frac{e_{xy}}{2}Y-\frac{e_{zx}}{2}Z\\ Y_{\text{ort}}=Y-\frac{e_{xy}}{2}X-\frac{e_{yz}}{2}Z\\ Z_{\text{ort}}=Z-\frac{e_{zx}}{2}X-\frac{e_{yz}}{2}Y \end{array}$$
3. Normalize columns to be unit vectors. Approximate formula (first order Taylor series) can be used only if normalization is performed incrementally by small steps (after each updating of the rotational matrix):
  (9) $$\begin{array}{c} X_{\text{norm}}=\frac{X_{\text{ort}}}{\left|X_{\text{ort}}\right|}\approx \frac{1}{2}\left(3-X_{\text{ort}}\cdot X_{\text{ort}}\right)X_{\text{ort}}\\ Y_{\text{norm}}=\frac{Y_{\text{ort}}}{\left|Y_{\text{ort}}\right|}\approx \frac{1}{2}\left(3-Y_{\text{ort}}\cdot Y_{\text{ort}}\right)Y_{\text{ort}}\\ Z_{\text{norm}}=\frac{Z_{\text{ort}}}{\left|Z_{\text{ort}}\right|}\approx \frac{1}{2}\left(3-Z_{\text{ort}}\cdot Z_{\text{ort}}\right)Z_{\text{ort}} \end{array}$$

Conversion from 3-2-1 Euler angles to the rotational matrix is given by the following formula [17]:

(10) $$R=R_{x}(\alpha )\cdot R_{y}(\beta )\cdot R_{z}(\gamma )=\left[\begin{array}{ccc} c_{\beta }c_{\gamma } & c_{\beta }s_{\gamma } & -s_{\beta }\\ s_{\alpha }s_{\beta }c_{\gamma }-c_{\alpha }s_{\gamma } & s_{\alpha }s_{\beta }s_{\gamma }+c_{\alpha }c_{\gamma } & s_{\alpha }c_{\beta }\\ c_{\alpha }s_{\beta }c_{\gamma }+s_{\alpha }s_{\gamma } & c_{\alpha }s_{\beta }s_{\gamma }-s_{\alpha }c_{\gamma } & c_{\alpha }c_{\beta } \end{array}\right]$$

where:

(11) $$\begin{array}{cc} c_{\alpha }=\cos\alpha & s_{\alpha }=\sin\alpha \\ c_{\beta }=\cos\beta & s_{\beta }=\sin\beta \\ c_{\gamma }=\cos\gamma & s_{\gamma }=\sin\gamma \end{array}$$

Conversion from the rotational matrix to 3-2-1 Euler angles can be done by the following algorithm:

(12) $$\begin{array}{ccc} \begin{array}{l} \text{if }R_{13}\leq -1:\\ \;\alpha =0\\ \;\beta =\pi /2\\ \;\gamma =-\text{atan2}\left(R_{21},R_{31}\right) \end{array} & \begin{array}{l} \text{else if }R_{13}\geq 1:\\ \;\alpha =0\\ \;\beta =-\pi /2\\ \;\gamma =\text{atan2}\left(-R_{32},R_{22}\right) \end{array} & \begin{array}{l} \text{else:}\\ \;\alpha =\text{atan2}\left(R_{23},R_{33}\right)\\ \;\beta =\arcsin\left(-R_{13}\right)\\ \;\gamma =\text{atan2}\left(R_{12},R_{11}\right) \end{array} \end{array}$$

The function atan2(y, x) is a four quadrant inverse tangent function, *i.e.*, arctangent function extended to the output angle interval from –π to π. Inputs x and y are coordinates of any point in 2D plane, output is an oriented angle between x-axis and the vector [x, y]. Function is supported by many programming languages by standard (e.g., C-language), having two arguments. The purpose of using two arguments instead of one is to gather information on the signs of the inputs in order to return the appropriate quadrant of the computed angle, which is not possible for the single-argument arctangent function.

### 1.3. Rotation around Arbitrary Axis

According to the Euler theorem it is possible to replace every rotation representation by simple rotation around angle *θ* around the arbitrary axis given by the unit vector ***n*** = ***n****'* = [*n_x_*, *n_y_*, *n_z_*] (length of the axis vector is |***n***| = 1). Note that the axis vector has the same coordinates in the inertial system *S* and the body-fixed system *S'*.

Transformation of the vector ***r*** from the system *S* to *S'* is expressed by the Rodriguez rotation formula:

(13) $$r^{\prime }=r+n\times \left[r\sin\theta +\left(n\times r\right)\left(1-\cos\theta \right)\right]$$

Inverse rotation is expressed by the identical axis ***n*** and opposite angle −*θ.* Chaining of two rotations around non-parallel axes of rotation is impossible to implement trivially, transformation to another type of expression is needed.

### 1.4. Quaternion

Quaternion (invented by sir William Rowan Hamilton in 1843) is a modification of rotation around arbitrary axis expression utilizing algebra of complex numbers expanded to three imaginary dimensions with the complex units *i*, *j*, *k*, for which it is valid:

(14) $$\begin{array}{ccc} i.i=-1 & i.j=k & i.k=-j\\ j.i=-k & j.j=-1 & j.k=i\\ k.i=j & k.j=-i & k.k=-1 \end{array}$$

Based on the expanded Euler’s formula, the rotation for quaternion around the axis $n=[n_{x},n_{y},n_{z}]$ by angle *θ* is defined as follows:

(15) $$q=\left(n_{x}.i+n_{y}.j+n_{z}.k\right)\sin\frac{\theta }{2}+\cos\frac{\theta }{2}=xi+yj+zk+w$$

While the axis ***n*** is a unit 3D vector, quaternion must follow unit constraint to be pure rotational:

(16) $$\left|q\right|=\sqrt{x^{2}+y^{2}+z^{2}+w^{2}}=1$$

Normalization of quaternion is done by the similar way like normalization of any vector. An approximate formula (like matrix normalization) can be used only if normalization is performed after each update of the quaternion:

(17) $$q_{\text{normalized}}=\frac{q}{\sqrt{x^{2}+y^{2}+z^{2}+w^{2}}}\approx \frac{\left(3-x^{2}-y^{2}-z^{2}-w^{2}\right)}{2}q$$

The advantage of quaternions is quick computing of chaining of rotation ***q***_1_ followed by ***q***_2_ utilizing Hamilton’s product:

(18) $$\begin{array}{c} q=q_{2}q_{1}=\left(x_{2}i+y_{2}j+z_{2}k+w_{2}\right)\left(x_{1}i+y_{1}j+z_{1}k+w_{1}\right)\\ =\left(x_{1}w_{2}+w_{1}x_{2}+z_{1}y_{2}-y_{1}z_{2}\right)i+\\ +\left(y_{1}w_{2}-z_{1}x_{2}+w_{1}y_{2}+x_{1}z_{2}\right)j+\\ +\left(z_{1}w_{2}+y_{1}x_{2}-x_{1}y_{2}+w_{1}z_{2}\right)k+\\ +\left(w_{1}w_{2}-x_{1}x_{2}-y_{1}y_{2}-z_{1}z_{2}\right) \end{array}$$

There are two basic variants of vector transformation utilizing quaternion. The first one takes the transformed vector as a quaternion $r=r_{x}i+r_{y}j+r_{z}k+0$:

(19) $$r^{\prime }={r^{\prime }}_{x}i+{r^{\prime }}_{y}j+{r^{\prime }}_{z}k=qrq^{-1}$$

Concerning speed it is better to use the following formula:

(20) $$r^{\prime }=r+2\hat{q}\times \left[rw+\left(\hat{q}\times r\right)\right]$$

where $\hat{q}$ is a vector part of quaternion:

(21) $$\hat{q}=\left[x,y,z\right]=n\sin\frac{\theta }{2}$$

Conversion from 3-2-1 Euler angles to unit quaternion is given by the following formula:

(22) $$q=\left[\begin{array}{c} x\\ y\\ z\\ w \end{array}\right]=\left[\begin{array}{c} s_{x}c_{y}c_{z}-c_{x}s_{y}s_{z}\\ c_{x}s_{y}c_{z}+s_{x}c_{y}s_{z}\\ c_{x}c_{y}s_{z}-s_{x}s_{y}c_{z}\\ -c_{x}c_{y}c_{z}-s_{x}s_{y}s_{z} \end{array}\right]\text{ where }\begin{array}{cc} s_{x}=\sin\left(\alpha /2\right) & c_{x}=\cos\left(\alpha /2\right)\\ s_{y}=\sin\left(\beta /2\right) & c_{y}=\cos\left(\beta /2\right)\\ s_{z}=\sin\left(\gamma /2\right) & c_{z}=\cos\left(\gamma /2\right) \end{array}$$

Conversion from quaternion to 3-2-1 Euler angles can be done by the following algorithm:

(23) $$\begin{array}{l} R_{13}=2\left(xz+yw\right)\\ \text{if }R_{13}\geq -1:\\ \;\alpha =0\\ \;\beta =\pi /2\\ \;\gamma =-\text{atan2}\left(xy+yz,\;xz-yw\right)\\ \text{else if }R_{13}\leq 1:\\ \;\alpha =0\\ \;\beta =-\pi /2\\ \;\gamma =\text{atan2}\left(-yz-xw,\;2-x^{2}-z^{2}\right)\\ \text{else:}\\ \;\alpha =\text{atan2}\left(yz-xw,\;2-x^{2}-y^{2}\right)\\ \;\beta =\arcsin\left(-R_{13}\right)\\ \;\gamma =\text{atan2}\left(xy-zw,\;2-y^{2}-z^{2}\right) \end{array}$$

## 2. Experimental Section

**Figure 3:**
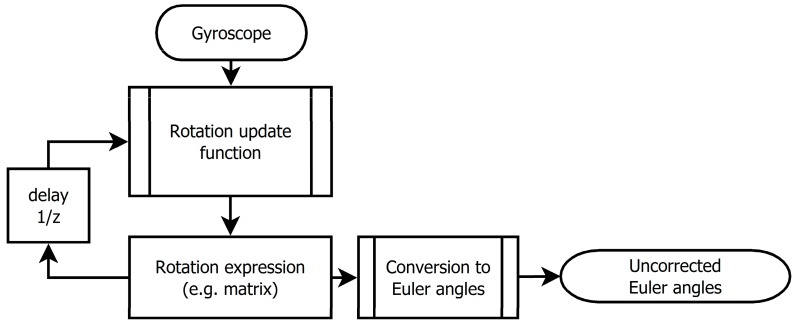
Schematics expressing principle of real-time gyroscope data processing.

In this section we compare errors of Euler angle estimation caused by algorithms processing gyroscopic data and being based on different rotation notations. These errors increase during run-time and depend on sampling frequency. The gyroscope firmly joined w ith the moving object *S'* is measuring angular velocity as a tri-component vector $\omega^{\prime }=[{\omega^{\prime }}_{x},{\omega^{\prime }}_{y},{\omega^{\prime }}_{z}]$. These data are sampled with given sample frequency *f*_sample_ = 1/Δ*T*. The sensor system has to process data sample by sample in real-time (Figure 3). As mentioned above, outputs of the algorithm are Euler angles *α*, *β*, *γ*, the system should also provide utility of the transformation of the vector from the *S* to *S'* coordinate system.

In order to eliminate influence of the sensor itself a model of the ideal digital-output gyroscope with the following properties was used for algorithm testing:

- Gyroscope output in each axis is a signed integer with 16-bit precision (like in many of available low-cost gyroscopes). Full-scale range of the output angular rate is ±500°/s.
- No noise is present at gyroscope output; also sampling frequency is absolutely precise (we want to examine errors of data processing algorithms, not precision of data itself). Therefore, data simulation was used instead of real experiment.

**Figure 4:**
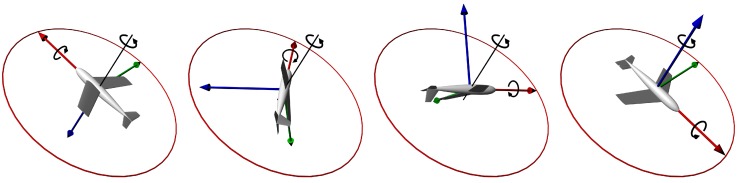
Half turn of the simulated precession movement.

In order to obtain comparable results, simulated movement of the object has to be exactly the same for all experiments. Therefore the pre-defined non-random movement has to be simulated. As a test input for algorithms we used a model of precession motion with perpendicular precession axis (see Figure 4). Such rotational movement is easy to define and also it is possible to analytically compute object’s attitude (Euler angles) at any time.

Angular velocity of primary rotation and precession was chosen *A* = 1 rad·s^−1^. Simulated angular velocity of the object (measured in its frame of reference) is then given by following:

(24) $${\omega^{\prime }}_{x}(t)=A;\;{\omega^{\prime }}_{y}(t)=A\sin\left(At\right);\;{\omega^{\prime }}_{z}(t)=A\cos\left(At\right)$$

**Figure 5:**
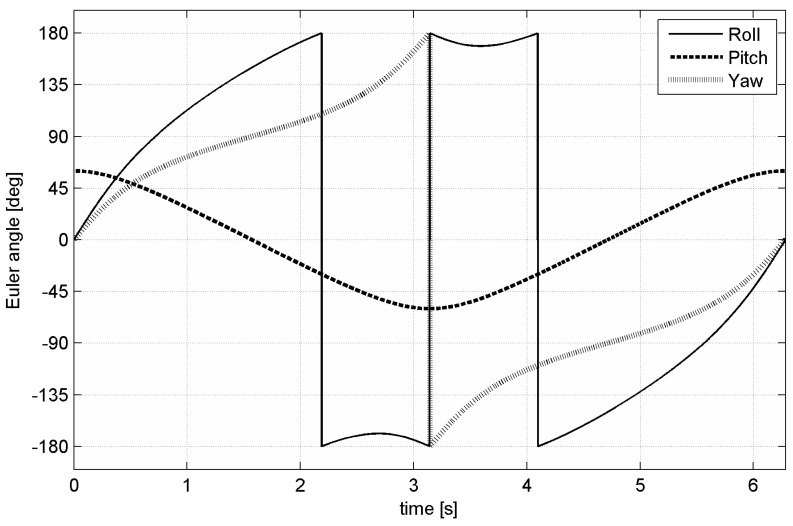
Euler angles during one turn of the simulated movement.

Simulation time corresponds to 20 turns (*t*_end_ = 40π/*A ≈* 2 min). Euler angles during simulated movement are shown in Figure 5. Initial rotation is {*α*_0_ = 0°, *β*_0_ = 60°, *γ*_0_ = 0°}. Euler angles (3-2-1 convention) during defined movement are given by following:

(25) $$\begin{array}{l} \alpha (t)=At+\text{atan2}\left(\sin\beta_{0}\sin At\;,\;\cos\beta_{0}\right)\\ \beta (t)=\arcsin\left(\sin\beta_{0}\cos At\right)\\ \gamma (t)=\text{atan2}\left(\sin At\;,\;\cos\beta_{0}\cos At\right) \end{array}$$

Resulting error of the algorithm is considered to be the maximal deviation of Euler angles $\tilde{\alpha }(t),\;\tilde{\beta }(t),\;\tilde{\gamma }(t)$ estimated by the algorithm from the correct Euler angles α(t),β(t),γ(t) during the simulation:

(26) $$err=\max\left(\left|\tilde{\alpha }(t)-\alpha \text{(}t)\right|,\;\left|\tilde{\beta }\text{(}t)-\beta \text{(}t)\right|,\;\left|\tilde{\gamma }\text{(}t)-\gamma \text{(}t)\right|\right)$$

Note that the difference between two angles has to be computed as angular difference (e.g., difference between 180° and −180° is zero) and the maximal shown error is 180°.

### 2.1. The Algorithm Based on Updating of the Rotational Matrix

**Figure 6:**
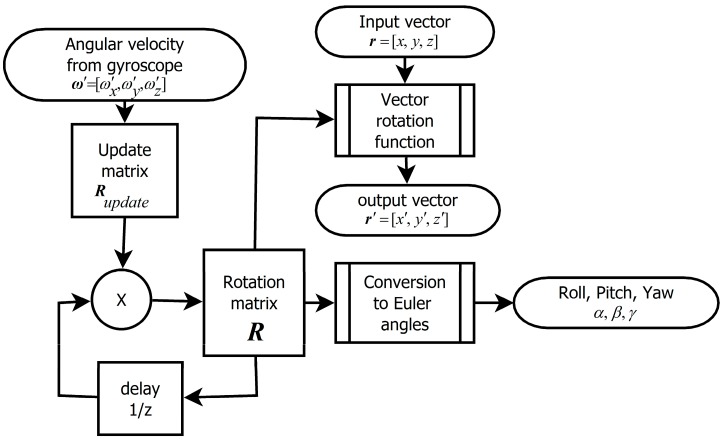
Precise version of the algorithm based on a rotation matrix.

The first version of the algorithm for processing of measured angular velocity is utilizing a matrix as a primary expression of rotation. The principle of this method is shown in Figure 6.

The original rotation matrix ***R****_n_* is multiplied by the update matrix ***R***_update_:

(27) $$R_{n+1}=R_{\text{update}}\cdot R_{n}$$

The update matrix defines rotation of the object between 2 recent samples of the angular velocity vector ***ω****'* (samples ***ω****_n_*_-1_ and ***ω****_n_*) with time span Δ*T*. It is possible to create the update matrix from angular velocity by two ways—precise and fast. Fast version uses linear approximation of sine and cosine functions which significantly reduces computational demands; precise version uses non-linear goniometric functions. There is a possibility of using Taylor series of higher order as an approximation of sine and cosine functions.

#### 2.1.1. Precise Version

We can assume that between 2 samples there is constant angular velocity, so its direction defines rotation axis and magnitude multiplied by sample period Δ*T* defines the angle of rotation:

(28) $$\begin{array}{l} n=\frac{\omega^{\prime }}{\left|\omega^{\prime }\right|}=\frac{\omega^{\prime }}{\sqrt{{\omega^{\prime 2}}_{x}+{\omega^{\prime 2}}_{y}+{\omega^{\prime 2}}_{z}}}=[n_{x},n_{y},n_{z}]\\ \theta =\left|\omega^{\prime }\right|\Delta T \end{array}$$

The corresponding update matrix is:

(29) $$R_{\text{update}}=\left[\begin{array}{ccc} c+{n_{x}}^{2}\left(1-c\right) & n_{x}n_{y}\left(1-c\right)+n_{z}s & n_{x}n_{z}\left(1-c\right)-n_{y}s\\ n_{y}n_{x}\left(1-c\right)-n_{z}s & c+{n_{y}}^{2}\left(1-c\right) & n_{y}n_{z}\left(1-c\right)+n_{x}s\\ n_{z}n_{x}\left(1-c\right)+n_{y}s & n_{z}n_{y}\left(1-c\right)-n_{x}s & c+{n_{z}}^{2}\left(1-c\right) \end{array}\right]$$

where c=cosθ and s=sinθ.

If we use substitution:

(30) $$\begin{array}{l} u=1-c\\ \begin{array}{ccc} u_{x}=n_{x}u & u_{y}=n_{y}u & u_{z}=n_{z}u \end{array}\\ \begin{array}{ccc} s_{x}=n_{x}s & s_{y}=n_{y}s & s_{z}=n_{z}s \end{array}\\ \begin{array}{ccc} r_{xx}=n_{x}u_{x} & r_{yy}=n_{y}u_{y} & r_{zz}=n_{z}u_{z} \end{array}\\ \begin{array}{ccc} r_{xy}=n_{x}u_{y} & r_{yz}=n_{y}u_{z} & r_{zx}=n_{z}u_{x} \end{array} \end{array}$$

We obtain:

(31) $$R_{\text{update}}=\left[\begin{array}{ccc} r_{xx}+c & r_{xy}+s_{z} & r_{zx}-s_{y}\\ r_{xy}-s_{z} & r_{yy}+c & r_{yz}+s_{x}\\ r_{zx}+s_{y} & r_{yz}-s_{x} & r_{zz}+c \end{array}\right]$$

#### 2.1.2. Fast Version

In case of high sampling frequency, we can use the infinitesimal rotation matrix based on the first order approximation of trigonometric functions:

(32) $$\underset{x\to 0}{\lim}\sin x=x\;\underset{x\to 0}{\lim}\cos x=1$$

The update matrix has a form of the infinitesimal rotational matrix:

(33) $$R_{\text{update}}=\left[\begin{array}{ccc} 1 & {\omega^{\prime }}_{z}dT & -{\omega^{\prime }}_{y}dT\\ -{\omega^{\prime }}_{z}dT & 1 & {\omega^{\prime }}_{x}dT\\ {\omega^{\prime }}_{y}dT & -{\omega^{\prime }}_{x}dT & 1 \end{array}\right]$$

Because of the linearity of equations (there is no need for calculation of trigonometric functions or normalization of the axis vector) this is the fastest of all mentioned methods (it is about 3-times faster than precise version, depending on the used hardware). However, the main disadvantage is low accuracy, which constrains this algorithm for systems with high sampling frequency.

**Figure 7:**
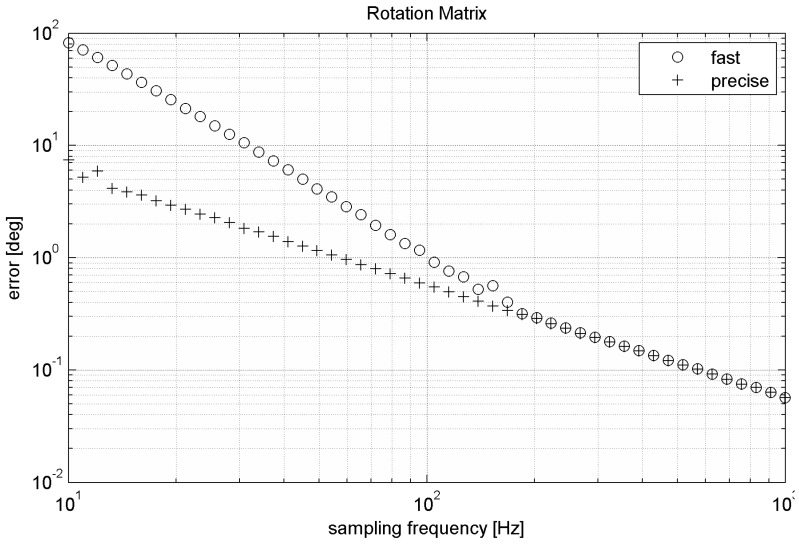
Errors of the matrix-based algorithms during simulated movement.

Figure 7 compares the fast and precise versions by their relative errors with respect to sampling frequency. Expression of rotation based on rotational matrices does not contain any singularities; therefore it is working with constant precision for every tilt. The advantage is also the quick algorithm of vector transformation. In order to maintain rotation matrix orthogonality, normalization is strongly recommended if fast version of the matrix-based algorithm is used. Shown results are computed after normalization in each step.

### 2.2. The Algorithm Based on the Integration of the Euler Angle Rates

**Figure 8:**
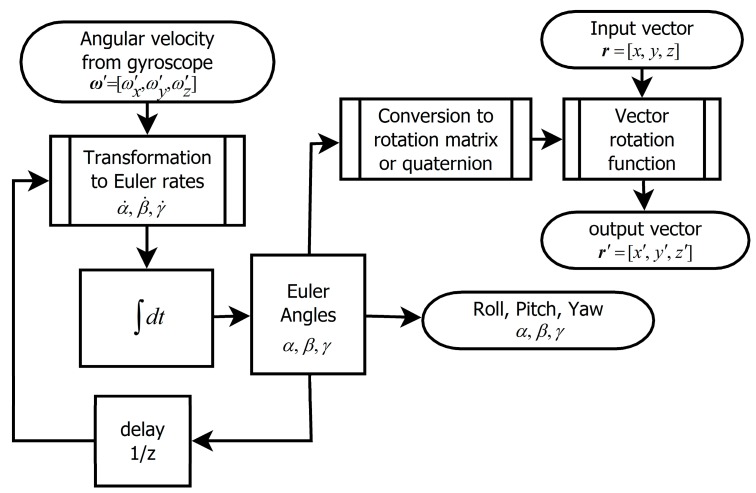
The algorithm based on Euler angle rates integration.

Using this algorithm it is possible to avoid intermediate expression of rotation (e.g., by the matrix) and following need for conversion to Euler angles. The principle is shown in Figure 8. This version uses relation between angular velocity ***ω****'* measured in the coordinate system *S'* and time derivations of Euler angles (Euler angle rates):

(34) $$\left[\begin{array}{c} \dot{\alpha }\\ \dot{\beta }\\ \dot{\gamma } \end{array}\right]=\left[\begin{array}{ccc} 1 & \frac{\sin\alpha \sin\beta }{\cos\beta } & \frac{\cos\alpha \sin\beta }{\cos\beta }\\ 0 & \cos\alpha & -\sin\alpha \\ 0 & \frac{\sin\alpha }{\cos\beta } & \frac{\cos\alpha }{\cos\beta } \end{array}\right]\cdot \left[\begin{array}{c} {\omega^{\prime }}_{x}\\ {\omega^{\prime }}_{y}\\ {\omega^{\prime }}_{z} \end{array}\right]$$

By integration of Euler angle rates $\dot{\alpha },\dot{\beta },\dot{\gamma }$ we get resulting Euler angles. There are two algorithms of numerical integration used in real-time processing:

Step integration:

(35) $$\alpha_{n+1}=\alpha_{n}+{\dot{\alpha }}_{n+1}\Delta T$$

Trapezoidal integration:

(36) $$\alpha_{n+1}=\alpha_{n}+\left({\dot{\alpha }}_{n-1}+{\dot{\alpha }}_{n}\right)\frac{\Delta T}{2}$$

**Figure 9:**
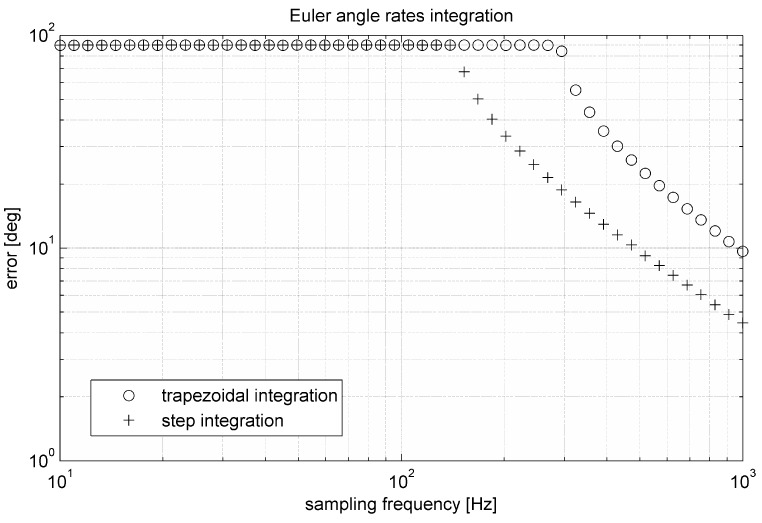
Relation between error and sampling frequency in the algorithm based on the integration of the Euler angle rates.

Although trapezoidal integration is usually more precise than simple step integration, according to Figure 9 step integration is in case of Euler angle rates little more precise. This is caused by non-linearity of transformation Equation (34). The algorithm is precise enough only at high sampling frequency.

**Figure 10:**
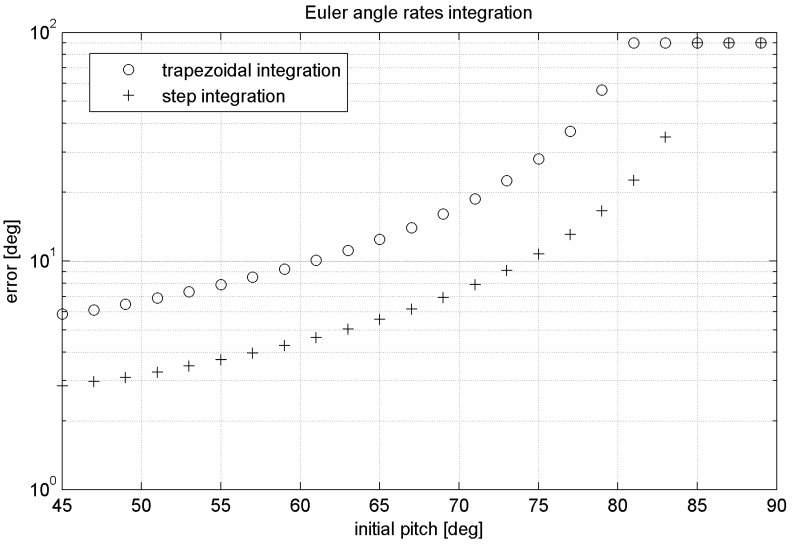
Relation between error and the initial pitch angle *β_0_* at *f*_sample_ = 1000 Hz of the Euler angle rates-based algorithm.

The main disadvantage of this algorithm is singularity of expression Equation (34) in case of cos*β* = 0 called gimbal-lock, which is representing the state, when *x*-axis is pointing downwards or upwards (*β* = 90° or *β* = −90° respectively). In surroundings of this singularity numerical error is rising. In case that position reaches this singularity, information about two DoF is lost (see Figure 10).

This error can be avoided by early conversion to another Euler convention which reaches singularity in other points (for example conversion to 1-2-1, 1-3-1, 2-3-1, 3-1-2, 3-1-3 or 3-2-3 Euler angle convention [18]). After calculation of Euler angles in substitute convention, they are transformed back to the primary convention. Accuracy is then achieved in the whole angle range. This is computation demanding non-linear operation [18].

### 2.3. The Algorithm Based on Quaternion

**Figure 11:**
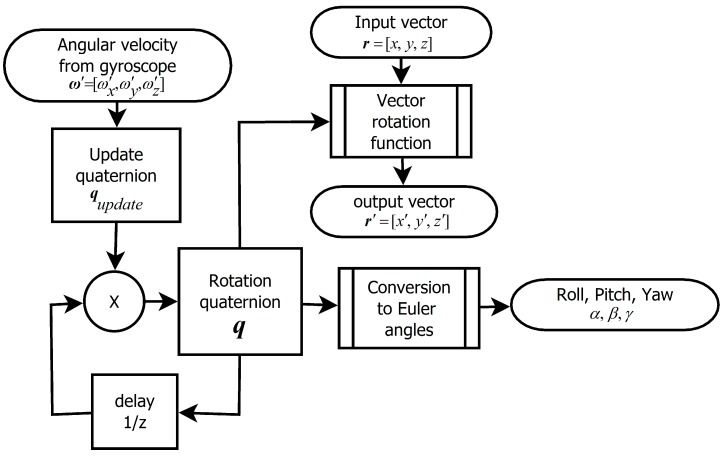
Principle of the quaternion-based algorithm.

The third possibility is to utilize primary expression of rotation using quaternion. The principle is expressed by Figure 11. Similarly as in the case of the rotational matrix, two variants of calculation are possible.

#### 2.3.1. Precise Version

It is an analogy of the precise matrix-based algorithm. The form of update quaternion is following:

(37) $$\begin{array}{c} q_{\text{update}}=\left({n^{\prime }}_{x}i+{n^{\prime }}_{y}j+{n^{\prime }}_{z}k\right)\sin\left(\frac{\omega^{\prime }\Delta T}{2}\right)+\cos\left(\frac{\omega^{\prime }\Delta T}{2}\right)\\ =\frac{\left({\omega^{\prime }}_{x}i+{\omega^{\prime }}_{y}j+{\omega^{\prime }}_{z}k\right)}{\omega^{\prime }}\sin\left(\frac{\omega^{\prime }\Delta T}{2}\right)+\cos\left(\frac{\omega^{\prime }\Delta T}{2}\right)\; \end{array}$$

where $\omega^{\prime }=\left|\omega^{\prime }\right|=\sqrt{{\omega^{\prime 2}}_{x}+{\omega^{\prime 2}}_{y}+{\omega^{\prime 2}}_{z}}$.

Then it is valid:

(38) $$q_{n}=q_{update}.q_{n-1}$$

#### 2.3.2. Fast Version

Neglecting higher order members, using approximations:

(39) $$\frac{{\omega^{\prime }}_{i}}{\omega^{\prime }}\sin\left(\frac{\omega^{\prime }\Delta T}{2}\right)\approx \frac{\omega^{\prime }\Delta T_{i}}{2}\;\cos\left(\frac{\omega^{\prime }\Delta T}{2}\right)\approx 1$$

We obtain update quaternion in the form:

(40) $$q_{\text{update}}=\left(\frac{{\omega^{\prime }}_{x}dt}{2}i+\frac{{\omega^{\prime }}_{y}dt}{2}j+\frac{{\omega^{\prime }}_{z}dt}{2}k+1\right)$$

Then according to Equation (38) it is valid:

(41) $$\begin{array}{c} q_{2}=q_{update}.q_{1}\\ \approx \left(\frac{{\omega^{\prime }}_{x}dt}{2}i+\frac{{\omega^{\prime }}_{y}dt}{2}j+\frac{{\omega^{\prime }}_{z}dt}{2}k+1\right)q_{1}\\ \approx q_{1}+\frac{dt}{2}\left({\omega^{\prime }}_{x}i+{\omega^{\prime }}_{y}j+{\omega^{\prime }}_{z}k\right)q_{1}=q_{1}+dq \end{array}$$

which results in:

(42) $$\frac{dq}{dt}=\frac{1}{2}\left({\omega^{\prime }}_{x}i+{\omega^{\prime }}_{y}j+{\omega^{\prime }}_{z}k\right)q$$

**Figure 12:**
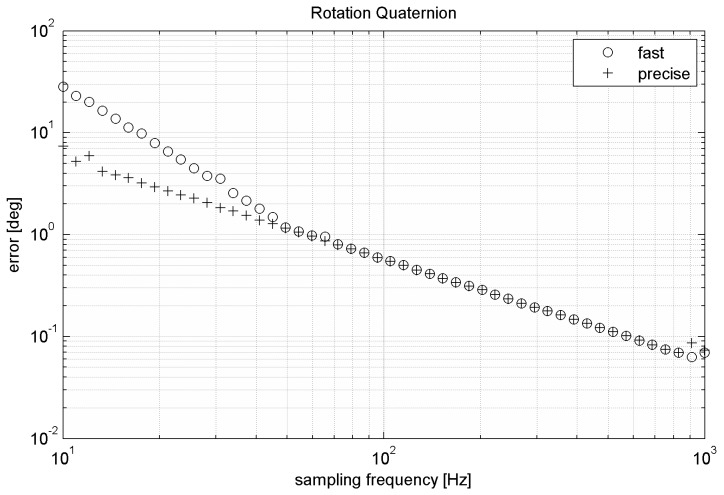
Errors of the quaternion-based algorithms during simulated movement.

By integration of quaternion derivation by time we get resulting rotation quaternion. Figure 12 compares precision of fast and precise versions of the algorithm. Like the fast matrix-based algorithm also the fast quaternion-based algorithm requires normalization of quaternion after each step. Normalization of rotation quaternion is described by Equation (17). Presented results are obtained with normalization.

### 2.4. Compensation of MEMS Gyroscope Data Using a MEMS Accelerometer and Magnetic Compass

Results given above are valid in an ideal case when gyroscope data are absolutely precise. Real MEMS gyroscope readings are noisy and sensitive to vibrations. The greatest impact on precision of Euler angles estimation has offset of the gyroscope. Due to variance of parameters of an electro-mechanical system with temperature the offset is also temperature dependent. The aim is to use secondary sensor (accelerometer, magnetic compass) to compensate increasing (offset-caused) error of the gyroscope-only system.

**Figure 13:**
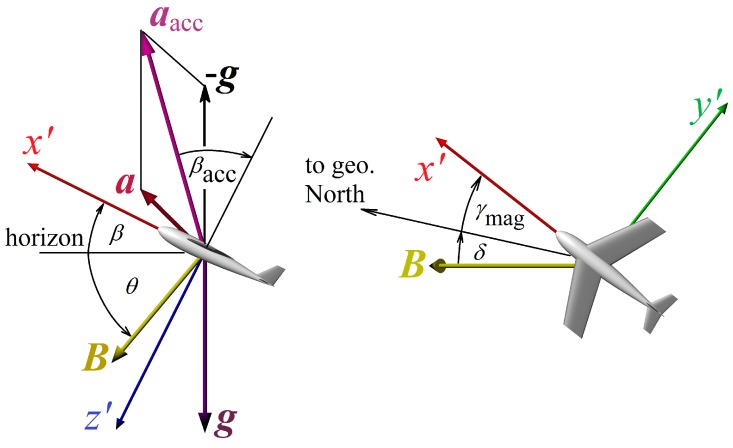
Accelerometer and magnetic compass readings at non-zero pitch *β* and yaw *γ*. Acceleration ***a***_acc_ is measured by the on-board accelerometer as a sum of the gravity acceleration ***g*** and object’s acceleration ***a***. Earth’s magnetic field induction ***B*** has inclination *θ*, declination *δ* and its horizontal complement points to magnetic North.

**Figure 14:**
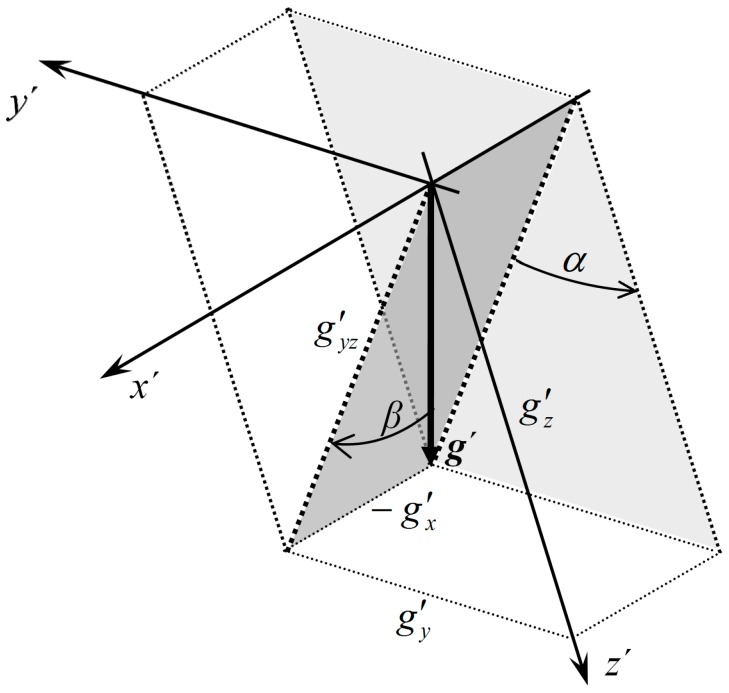
Roll and pitch calculation from measured gravity acceleration. Object pitches up and rolls right (axis *x'* points forward). The vector ***g****'* defines vertical direction.

The accelerometer is sensing its acceleration (3D vector) relative to inertial frame of reference. In gravitational field the accelerometer is sensing gravity as acceleration upwards. Reading of the accelerometer is (see Figure 13 and Figure 14):

(43) $$a_{\text{acc}}=a^{\prime }-g^{\prime }+a_{\text{noise}}=[a_{\text{acc}X},a_{\text{acc}Y},a_{\text{acc}Z}]$$

where ***a****'* is own acceleration of the object expressed in the coordinate system *S'*, ***g****'* is a vector of gravitational acceleration (depending on locality near Earth) transformed to the coordinate system of the object *S'* based on data concerning object rotation and ***a***_noise_ is the noise caused by:

• Vibrations of this object
• Thermal noise of the sensor
• Quantization noise of the A/D converter

According to Figure 14 for roll and pitch angle we obtain:

(44) $$\alpha_{\text{acc}}=\text{atan2}({g^{\prime }}_{y},\;{g^{\prime }}_{z})\approx \text{atan2}(-a_{\text{accY}},\;-a_{\text{accZ}})$$

(45) $$\beta_{\text{acc}}=\text{atan2}(-{g^{\prime }}_{x},\;{g^{\prime }}_{yz})=\text{atan2}(-{g^{\prime }}_{x},\;\sqrt{{g^{\prime 2}}_{y}+{g^{\prime 2}}_{z}})\approx \text{atan2}(a_{\text{accX}},\;\sqrt{{a_{\text{accY}}}^{2}+{a_{\text{accZ}}}^{2}})$$

If we assume that noise ***a***_noise_ has zero mean value and lower limiting frequency *f*_min_, then the noise can be effectively suppressed by the low pass filter.

Since we cannot determine the rotation around vertical *z'*-axis (yaw *γ*) from accelerometer data, it is necessary to add a magnetic compass to the sensor system. For ensuring proper function of the system for all rotations of the object, the magnetic sensor has to determine magnetic induction ***B****'* of the Earth’s magnetic field in all three axes (compass output is the vector $B^{\prime }=[{B^{\prime }}_{x},{B^{\prime }}_{y},{B^{\prime }}_{z}]$). For yaw rotation calculated from readings of the magnetic sensor it is valid:

(46) $$\gamma_{\text{mag}}=\text{atan2}(-B_{y1},B_{x1})-\delta_{m}$$

where *δ*_m_ is magnetic declination (offset between magnetic and geographic north direction, depending on actual position on Earth), *B_x_*_1_ and *B_y_*_1_ are components of measured magnetic induction after transformation to the coordinate system *S*_1_ (inverted *x*- and following *y*- rotation, see definition of Euler angles) according to the formula:

(47) $$B_{1}=[B_{x1},B_{y1},B_{z1}]={ℜ}_{\alpha ,\beta }^{-1}(B^{\prime })$$

In terms of avoiding preparation of partial inverse rotation, it is more convenient to determine the difference between yaw *γ*_gyro_ calculated from gyroscope data and yaw from the magnetic compass *γ*_mag_ as:

(48) $$\Delta \gamma_{\text{mag}}=\gamma_{\text{mag}}-\gamma_{\text{gyro}}=\text{atan2}(-B_{y},B_{x})-\delta_{m}$$

where *B_x_* a *B_y_* are components of measured magnetic induction after transformation to the coordinate system *S* (inverted *x*-, *y*- and *z*- rotation), which are:

(49) $$B=[B_{x},B_{y},B_{z}]={ℜ}_{\alpha ,\beta ,\gamma }^{-1}(B^{\prime })$$

**Figure 15:**
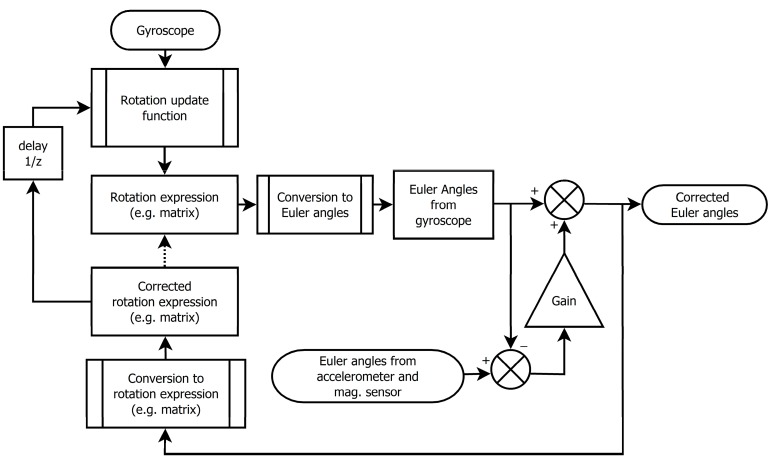
Data fusion of the gyroscope, accelerometer and magnetic sensor.

For fusion of Euler angles measured by the gyroscope as a primary sensor and accelerometer and magnetic compass as secondary sensors we can use the algorithm as shown in Figure 15. Gain *K* << 1 expresses relative weight of the accelerometer with respect to the gyroscope (if *K* = 0, the accelerometer does not affect output Euler angles). Delay block and gain forms the first order discrete low pass filter in the accelerometer signal path with cutoff frequency:

(50) $$f_{\max}\approx Kf_{\text{sample}}$$

The fusion schema does not filter out any noise from gyroscope reading; it suppresses increasing error of estimated Euler angles in long term caused mainly by offset.

While the schematics in Figure 15 contains the reverse conversion block from Euler angles to the rotation matrix, normalization of the matrix is no longer needed.

## 3. Results and Discussion

**Figure 16:**
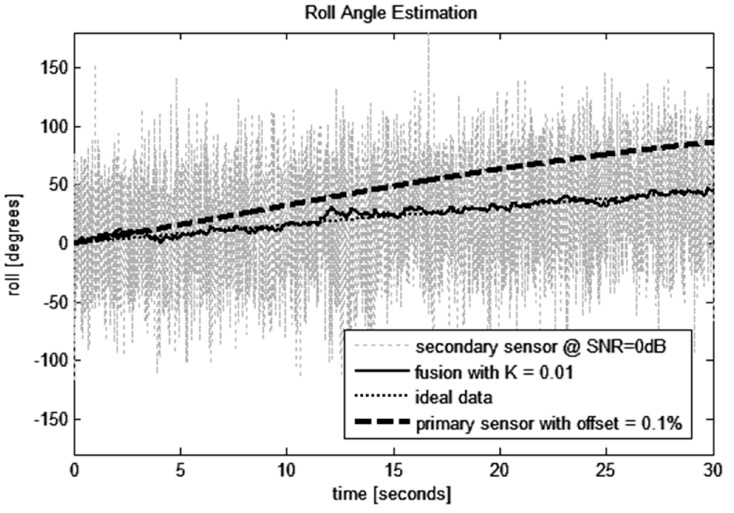
Estimation of roll angle with gyroscope offset 0.1% of full range (500°/s).

Effect of sensor fusion is more significant after longer time (especially at low angular velocities). Figure 16 shows effect of using fusion of gyroscope, accelerometer and magnetic sensor readings. Simulated rotation was slowed down 100-times (*A* = 0.01, compare with Equation (24)). Fusion gain was *K* = 0.01, noise in secondary sensor data has SNR = 0dB. The precise quaternion-based algorithm at sampling frequency 1 kHz was used. Due to gyroscope offset the estimation error continuously increases with time. The low pass filter within the data fusion algorithm suppresses noise in secondary sensor data and roll angle obtained by fusion slightly oscillates around actual roll.

**Figure 17:**
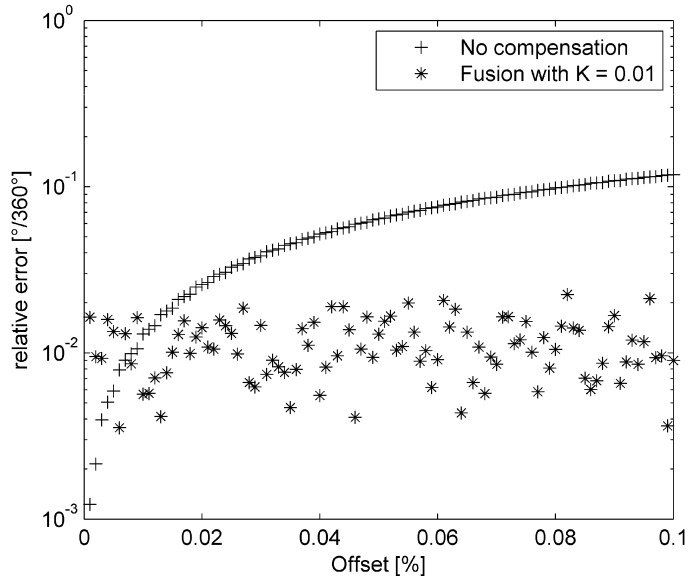
Relative error of roll angle estimation with respect to gyroscope offset.

As can be seen in Figure 17, sensor fusion with weak bound of secondary absolute but noisy sensor can effectively suppress error of estimation caused by sensor offsets. Fusion gain *K* has to be set according to offset variance of the gyroscope (the more precise the gyroscope is the lesser fusion gain can be obtained).

The second great aspect of the algorithm is its computational time. Two types of reference hardware were used:

- 8-bit low-cost microprocessor (Atmel ATmega1284P running at 20 MHz);
- 32-bit microprocessor with FPU and DSP support (Atmel UC3C1512C running at 48 MHz).

Table 1 compares computational time of algorithms in terms of the CPU cycles of 8-bit low-cost microprocessor. The mentioned cycle counts are average values from 1000 random inputs, using the mathematical library optimized for AVR 8-bit microcontrollers. Algorithms are using software-implemented single precision floating point arithmetic (according to IEEE 754) due to the fact that AVR microcontrollers do not contain the floating point unit (FPU). Using highly optimized implementation of the matrix-based algorithm including fusion of the gyroscope with accelerometer and magnetic compass allows algorithm sampling rate up to approximately 200 Hz (running on AVR 8-bit core @ 20 MHz).

Table 2 shows the same algorithms running on the 32-bit microprocessor. Utilization of the 32-bit microcontroller with FPU significantly reduces the count of needed clock cycles (in case of adding and multiplication of real numbers approx. 30 times depending on the used processor). While the representation of numbers is the same for all architectures (32-bit floating point number), accuracy of the algorithm does not depend directly on the used microcontroller. However, decreasing time needed for one cycle of the algorithm allows higher maximal sample rate (up to maximal sample rate of gyroscope itself). Increasing sample rate will improve accuracy significantly. For example, increasing sampling rate from 200 Hz to 1 kHz will decrease error caused by the algorithm by approx. 50% (see Figure 5, Figure 7 and Figure 10).

**Table 1:** Comparison of methods in terms of 8-bit AVR processor clock cycles.

Algorithm	Updating of the Rotational Matrix	Integration of the Euler Angle Rates	Updating of the Quaternion
Redundancy (count of variables)	**9	****3	***4
Gyroscope data processing (rotation update)	*** ^+^ 17,230 (6034 ^+^)	***14,750	****11,462 (5120 ^+^)
Normalization	**12,265	*****0	****1972
Vector transformation	****2301	*15,231 ^3^)	***4321
Transformation to the rotational matrix	*****0	**12,930	****3536
Transformation to Euler angles	****7820	*****0	***10,673
Transformation to quaternion	***3370	**13,020	*****0
Clock cycles for the gyroscope-only system ^1^)	37,315 (26,119 ^+^)	14,750	24,107 (17,765 ^+^)
Clock cycles for the compensated system ^2^)	40,281 (29,085 ^+^)	29,981 ^3^)	39,476 (33,134 ^+^)

Legend: ^+^ Fast version of the algorithm; * Improper; ** Usable; *** Good; **** Excellent; ***** No demands on computing time; ^1^) Cycles needed for gyroscope data processing, normalization (if needed) and conversion to Euler angles; ^2^) Cycles needed for gyroscope data processing, transformation of the magnetic induction vector (compass), conversion to Euler angles and back; ^3^) Euler angles were converted to the rotational matrix which was used for vector transformation.

**Table 2:** Comparison of methods in terms of 32-bit UC3C processor clock cycles.

Algorithm	Updating of the Rotational Matrix	Integration of the Euler Angle Rates	Updating of the Quaternion
Gyroscope data processing (rotation update)	***4511 (247 ^+^)	**10,900	****4219 (169 ^+^)
Normalization	**226	*****0	****66
Vector transformation	****58	*13,757 ^3^)	***182
Transformation to the rotational matrix	*****0	**13,669	****752
Transformation to Euler angles	****10,014	*****0	***10,648
Transformation to quaternion	****1049	**11,337	*****0
Clock cycles for the gyroscope-only system ^1^)	14,751 (10487 ^+^)	10,900	14,933 (10,883 ^+^)
Clock cycles for the compensated system ^2^)	28,252 (23988 ^+^)	24,657 ^3^)	26,386 (22,336 ^+^)

Legend: ^+^ Fast version of the algorithm; * Improper; ** Usable; *** Good; **** Excellent; ***** No demands on computing time; ^1^) Cycles needed for gyroscope data processing, normalization (if needed) and conversion to Euler angles; ^2^) Cycles needed for gyroscope data processing, transformation of the magnetic induction vector (compass), conversion to Euler angles and back; ^3^) Euler angles were converted to the rotational matrix which was used for vector transformation.

If the microcontroller with hardware support of floating-point calculations is used, linear (fast) versions of algorithms are much faster than the precise non-linear algorithms. Results given in Table 1 and Table 2 strongly depend on implementation of the discussed algorithms (execution speed can be improved by using optimized mathematical libraries for hardware supporting floating-point calculations). Number of the clock ticks is shown mainly for simple comparison purposes.

The Euler angle rates integration can be faster than the remaining two algorithms but it has significantly worse accuracy at the same sampling frequency and also has intrinsic singularity. Therefore the choice should be between the matrix- and quaternion-based algorithms. If the sensor system should be able to quickly transform many vectors between inertial and local frame of reference the matrix-based algorithm can be a better choice (2–3 times faster vector transformation than by quaternion).

## 4. Conclusions

By comparing relative errors of each mentioned algorithm we can see that the worst algorithm is direct Euler angles integration due to its singularity. Precise version of the quaternion-based algorithm is slightly faster than the precise matrix-based algorithm. Fast version of the quaternion-based algorithm at lower sampling frequency is also more accurate than the matrix-based algorithm (see Table 3). Difference in accuracy between fast and precise versions of the same algorithm decreases with sampling frequency (see Figure 7 and Figure 12). The choice of the proper algorithm depends on:

- Available computational power (CPU) and maximal sampling frequency of the sensors (which reflects in overall cost of the sensor system and its accuracy). At lower sampling frequency the fast quaternion-based algorithm is more precise than the fast matrix-based algorithm.
- Precision requirements (in order to achieve long-term stability the compensated system with sensor fusion has to be used).
- Amount of vectors transformed from the non-rotated coordinate system to rotated coordinates and vice versa (transformation performed by the matrix is faster).

**Table 3:** Accuracy of the algorithms.

Sampling Frequency	Maximal Error of the Algorithm during 120 s of Simulated Movement				
	Matrix-Based Algorithm		Integration of Euler Angle Rates	Quaternion-Based Algorithm	
	Fast	Precise	Step Integration	Fast	Precise
10 Hz	>180°	8°	>180°	30°	8°
50 Hz	4°	1°	>180°	1°	1°
100 Hz	1°	0.6°	>180°	0.6°	0.6°
500 Hz	0.1°	0.1°	8°	0.1°	0.1°
1000 Hz	0.06°	0.06°	4°	0.06°	0.06°
